# Layer-by-Layer Immobilization of DNA Aptamers on Ag-Incorporated Co-Succinate Metal–Organic Framework for Hg(II) Detection

**DOI:** 10.3390/s24020346

**Published:** 2024-01-06

**Authors:** Shubham S. Patil, Vijaykiran N. Narwade, Kiran S. Sontakke, Tibor Hianik, Mahendra D. Shirsat

**Affiliations:** 1 RUSA-Centre for Advanced Sensor Technology, Department of Physics, Dr. Babasaheb Ambedkar Marathwada University, Aurangabad 431004, India; shubhamspatil10297@gmail.com (S.S.P.); vkiranphysics@gmail.com (V.N.N.); 2 Department of Nuclear Physics and Biophysics, Faculty of Mathematics, Physics and Informatics, Comenius University, 842 48 Bratislava, Slovakia; kiransontakke07@gmail.com

**Keywords:** DNA aptamers, metal–organic frameworks, layer-by-layer immobilization, electrochemical sensors, heavy metal ions

## Abstract

Layer-by-layer (LbL) immobilization of DNA aptamers in the realm of electrochemical detection of heavy metal ions (HMIs) offers an enhancement in specificity, sensitivity, and low detection limits by leveraging the cross-reactivity obtained from multiple interactions between immobilized aptamers and developed material surfaces. In this research, we present a LbL approach for the immobilization of thiol- and amino-modified DNA aptamers on a Ag-incorporated cobalt-succinate metal–organic framework (MOF) (Ag@Co-Succinate) to achieve a cross-reactive effect on the electrochemical behavior of the sensor. The solvothermal method was utilized to synthesize Ag@Co-Succinate, which was also characterized through various techniques to elucidate its structure, morphology, and presence of functional groups, confirming its suitability as a host matrix for immobilizing both aptamers. The Ag@Co-Succinate aptasensor exhibited extraordinary sensitivity and selectivity towards Hg(II) ions in electrochemical detection, attributed to the unique binding properties of the immobilized aptamers. The exceptional limit of detection of 0.3 nM ensures the sensor’s suitability for trace-level Hg(II) detection in various environmental and analytical applications. Furthermore, the developed sensor demonstrated outstanding repeatability, highlighting its potential for long-term and reliable monitoring of Hg(II).

## 1. Introduction

Heavy metal ions (HMIs) are pervasive environmental pollutants known for their detrimental health effects and ecological consequences [1,2]. Heavy metals can enter the environment through industrial discharges, agricultural runoff, mining, and even natural erosion, and they tend to accumulate in soils, water bodies, and the food chain [3,4,5]. This persistence can lead to the bioaccumulation of heavy metals in living organisms, posing severe health risks to humans and wildlife. The development of advanced and sensitive detection methods for HMIs is of paramount importance to mitigate these concerns. Electrochemical sensors, with their high sensitivity and selectivity, have emerged as a promising avenue for HMI detection [6].

MOFs as electrode modifiers enhance HMI detection due to high surface area, tunable pores, and functionalizable structures [7,8,9,10,11,12]. MOFs offer a versatile and promising platform for HMI detection, contributing to environmental protection, public health, and safety by providing highly sensitive, selective, and efficient detection methods. Researchers continue to explore and develop new MOF-based materials and sensor designs to address the challenges of HMIs contamination. Various approaches have been explored before to inculcate the conductivity of the MOFs [13,14]. The incorporation of metal nanoparticles into an MOF array is one of the most prominent approaches to tune the conductivity of the MOF. MOFs modified with specific metal nanoparticles have shown great promise in HMI detection. These modified MOFs leverage the properties of both the MOFs and the incorporated metal nanoparticles to enhance the selectivity, sensitivity, and versatility of HMIs sensors. When managed appropriately, cross-reactivity in electrochemical detection can offer several positive advantages: enhanced sensitivity, reduced false negatives, multiplexed detection, etc. Even though cross-reactivity has numerous benefits, it should be carefully managed, validated, and controlled to avoid issues such as false positives, loss of specificity, and inaccurate quantification [15]. The advantages of cross-reactivity can be harnessed effectively when the recognition elements and assays are thoughtfully designed for the intended application. Utilizing various MOFs, their composites with metal nanoparticles, and DNA aptamers as an immobilizer, various practices have been explored previously to reduce cross-reactivity instead of inculcating its benefits [16]. Several modifications in the aptamer immobilization methodologies prevent cross-reactive effects on HMI detection adopted by various research groups [17,18,19]. However, no single attempt has been made to govern the impact of cross-reactivity on the electrochemical detection of HMIs.

The layer-by-layer (LbL) immobilization of biorecognition elements on nanomaterial surfaces is a well-established strategy in the field of biosensors and analytical chemistry [20]. This method employs dual biorecognition layers on the electrode surface, boosting selectivity and sensitivity. Each layer enhances specificity, introducing cross-reactivity that amplifies the analytical signal for cross-reactive HMIs more than the targeted one, by improving the binding properties of each aptamer layer [21]. Cross-reactivity also improves sensitivity by amplifying analytical responses for cross-reactive HMIs. In this study, we proffer an innovative methodology encompassing the LbL immobilization of thiol- (Apt-SH) and amino-modified aptamers (Apt-NH) onto the intricate surface matrix of silver nanoparticle-incorporated cobalt succinate MOFs (Ag@Co-Succinate).

DNA or RNA aptamers are short single-stranded nucleotides with highly specific target binding capabilities. They have gained significant attention as versatile HMIs recognition in sensor development [22,23]. Through LbL immobilization of two distinct aptamers, we seek to leverage the combined selectivity and sensitivity of these receptors for the precise detection of the target analyte [24]. To govern extensive cross-reactivity for electrochemical detection of HMIs, we proposed novel LbL immobilization of Apt-SH and Apt-NH onto the Ag@Co-Succinate. Firstly, Co-Succinate MOF was synthesized by a hydrothermal method and then chemically modified with Ag nanoparticles (AgNPs) to enhance its conductivity. The first layer of immobilization involves Apt-SH, which carries thiol functional groups for covalent attachment to the MOF surface [25]. This initial layer provides selectivity for one class of target analytes and forms a robust foundation. Apt-NH is then immobilized in a second layer, introducing an additional dimension of selectivity by targeting a different class of analytes. This approach shows great potential in situations where multiple analytes are present, often with varying chemical structures, and requires the accurate and simultaneous detection of a range of targets. The LbL immobilization of Apt-SH and Apt-NH on Ag@Co-Succinate MOF provides a versatile platform for detecting tailored HMIs, which can broaden the scope of analyte detection and increase the specificity and sensitivity of the detection system. This research sets the stage for the development of advanced HMIs sensors with a wide range of applications, including environmental monitoring, clinical diagnostics, and food safety assessment.

## 2. Materials and Methods

### 2.1. Chemicals and Reagents

Cobalt nitrate hexahydrate (Co(NO_3_)_3_·6H_2_O) and silver nitrate (Ag(NO_3_)) were purchased from Sigma Aldrich (Darmstadt, Germany). Succinic acid (C_4_H_6_O_4_) and N,N-dimethylformamide (HCON(CH_3_)_2_) (DMF) were purchased from Alfa Aesar (Waltham, MA, USA). Deionized water (DI) was utilized as a solvent. As a precondition for making buffer solutions, sodium acetate (CH_3_-COO-Na), acetic acid (CH_3_COOH), sodium dihydro phosphate (NaH_2_PO_4_), potassium ferricyanide (K_3_[Fe(CN)_6_]), and sodium phosphate dibasic (Na_2_HPO_4_) were purchased from Molychem, Mumbai, India. Hg(II), Cd(II), Pb(II), Cu(II), Zn(II), Fe(II), and other HMI solutions were prepared by dissolving heavy metal chloride salts in DI water. In brief, a 1 M stock solution was prepared for each heavy metal by adding the necessary weight (in milligrams) of HMI salts in the desired volume (100 mL). Following the preparation of the stock solution, the concentration reduction procedure was used to generate a concentration of HMIs, ranging from 0.7 nM to 10 nM. DNA aptamers (5′-TTT TTT ACC CAG GGT GGG TGG GTG GGT-3′) [26] modified at 5′ terminal by thiol (Apt-SH) or amino-linker (Apt-NH) were purchased from GeneCust (Boynes, France).

### 2.2. Synthesis of Co-Succinate and Ag@Co-Succinate

With some adjustments made in accordance with previous literature [27], the solvothermal method was utilized to synthesize Co-succinate [27]. In a combination of 50 mL N,N-dimethylformamide (DMF) and 2.5 mL DI solvents, approximately 1.5 mmol of the 1.71 g succinic acid ligand was dissolved. The blend was swirled for 10 min. After that, the mixture was agitated for a further half hour while 1 mmol of Co(NO_3_)_2_ was added. The reaction mixture was subsequently heated at 120 °C for 20 h. Co-MOF produced purple crystals as a byproduct, which were separated and repeatedly cleaned using a 5:0.25 DMF and DI mixture. The resulting goods were subsequently heated at 60 °C to dry them out.

**Scheme 1:**
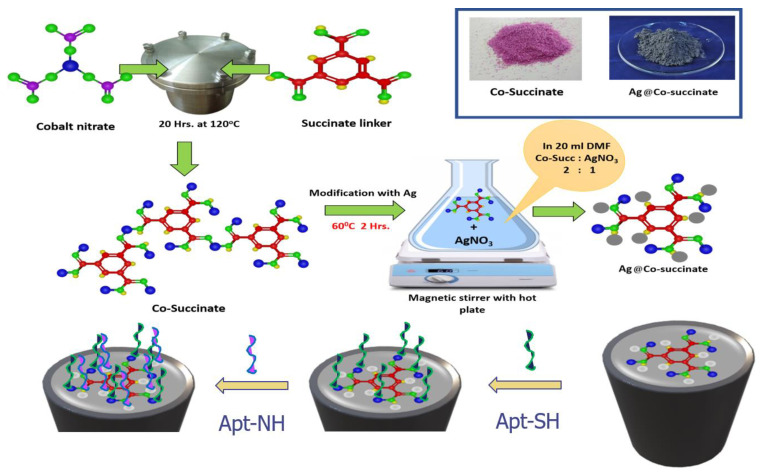
Graphical representation of synthesis of Co-Succinate, Ag incorporation into Co-Succinate, and LbL immobilization of Apt-SH and Apt-NH of Ag@Co-Succinate.

Moreover, 0.5 g of Ag(NO)_3_ salt in proportion to Co-Succinate was used to modify each metal. The mixture was placed in 20 mL of DMF and annealed for 5 h at 60 °C. The brown-colored precipitate was dried using a vacuum filter and dried overnight at room temperature. The process of synthesis of Co-Succinate and Ag@Co-Succinate is illustrated in Scheme 1.

### 2.3. Characterization Techniques

An X-ray diffractometer (XRD) 40.0 mA and 40.0 kV with a monochromatic CuK radiation source (λ = 1.54 Å) (Bruker D8 Advance, Bremen, Germany) was used for structural characterization of synthesized materials. Fourier-Transformed Infrared spectroscopy (FTIR) was taken with the Bruker Alpha model’s ECO-ATR mode, which had an operating range of 600 cm^−1^ to 3000 cm^−1^ (Bruker, Bremen, Germany), and a Raman spectrophotometer (Xplora plus, Horiba Scientific, Paris, France) with a laser at 532 nm with an 1800 nm grating were used for spectroscopic characterization. Field emission scanning electron microscopy (FE-SEM) images of the materials were acquired using a TESCAN LYRA 3 (TESCAN, Brno, Czech Republic) operating at 30 kV. The electrochemical potentiostat and galvanostat CH-660C (CH-Insturments, Austin, TX, USA) was used for electrochemical characterization and electrochemical sensing.

### 2.4. Immobilization of DNA Aptamers on Ag@Co-Succinate

Firstly, 20 mg of Ag@Co-succinate was dispersed in 0.5 mL of DI. The suspended solution was prepared in a 1 mL Eppendorf tube. Then, 10 µL Nafion (Sigma-Aldrich, Saint Louis, MO, USA) was diluted in 1 mL of DI, and 5 µL of this diluted Nafion solution was added to an Eppendorf tube containing Ag@Co-Succinate. After 10 min. of ultrasonication, 5 µL of the above solution was drop-casted on a glassy carbon electrode (GCE) (CH Instruments, Austin, TX, USA). From 10 µM stock solution of the Apt-SH and Apt-NH, they were kept in small Eppendorf tubes to incubate in hot water around 95 °C until room temperature. Firstly, Ag@Co-Succinate modified GCE was incubated in Apt-SH for 5 h. And later on, electrodes were incubated with Apt-NH. Successive immobilization of Apt-SH and Apt-NH leads to the fabrication of Apt-SH-NH-Ag@Co-Succinate modified GCE electrode. LbL immobilization of Apt-SH and Apt-NH of Ag@Co-Succinate is illustrated in Scheme 1.

### 2.5. Preparation of the Aptasensors and Their Electrochemical Characterization

GCE, used for aptasensor preparation, was pre-treated by polishing it with 0.3 and 0.05-micron alumina slurry and sonicated in DI and ethanol for 2 min. and dried at room temperature. A total of 10 µL of each material (i.e., Co-Succinate, Ag@Co-Succinate) was cast over a 3 mm diameter GCE and left to dry at room temperature for 5 h. Also, the Apt-SH and Apt-NH were immobilized on Ag@Co-Succinate to determine their electrochemical response. A modified GCE was used as a working electrode, Ag/AgCl as a reference electrode, and platinum as a counter electrode. Differential pulse voltammetry (DPV) was used for the electrochemical detection of HMIs. In total, 10 mM phosphate-buffered saline (PBS), pH 7.4, was used as the electrolyte solution for setting up electrochemical cells. HMI solutions of varying concentrations ranging from 0.7 nM to 10 nM were introduced to an electrolyte solution. The selectivity measurements were performed using a constant concentration range of each HMI, i.e., 10 nM. Before the electrochemical sensing study, 0.1 M PBS containing 5 mM Fe(CN)_6_^3−/4−^ was used to record cyclic voltammograms (CVs). CV cycles were recorded at a scan rate of 0.1 V/s. Using an AC excitation signal and an open circuit potential of 300 mV in 10 mM PBS, electrochemical impedance spectroscopy (EIS) measurements were taken throughout a frequency range of 0.01 Hz to 100 kHz.

## 3. Results Discussion

### 3.1. MOFs Characterizations

#### 3.1.1. Structural, Spectroscopic, and Morphological Characterizations

**Figure 1:**
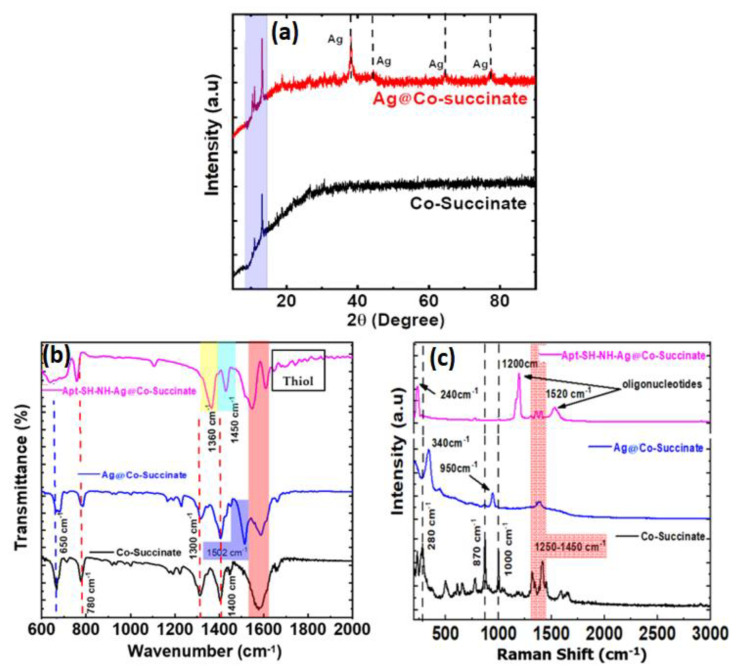
(**a**) X-ray diffraction patterns of Co-Succinate and Ag@Co-Succinate; (**b**) FTIR spectra of Co-Succinate, Ag@Co-Succinate, Apt-SH, and Apt-NH-Ag@Co-Succinate; (**c**) Raman spectra of Co-Succinate, Ag@Co-Succinate, Apt-SH, and Apt-NH-Ag@Co-Succinate.

The XRD pattern in Figure 1a shows the Co-Succinate material’s crystal lattice diffraction peaks at 2θ angles of 11.056° and 13.16°. These peaks are consistent with X-ray diffraction by the Co-Succinate crystal lattice. The pattern closely matches previously reported in the literature [27], indicating the synthesized Co-Succinate material’s reliability and consistency. The observed sharp diffraction peaks at 2θ angles of 38.15°, 44.33°, 64.5°, and 77.43°, and their similarity to the JCPDS (Joint Committee on Powder Diffraction Standards) card No. 04-0783, confirm the presence of Ag nanoparticles in the Co-Succinate [28,29]. The excessive peaks suggest the extensive presence of silver in the Ag@Co-Succinate without affecting the Co-Succinate’s phase. This means that the active sites for organic linkers remained unbonded, allowing specific HMIs to form a bond with these sites, as further explained by spectroscopic analysis.

The FTIR spectra of Co-Succinate and Ag@Co-Succinate in the range of 600 cm^−1^ to 2000 cm^−1^ are shown in Figure 1b. The peaks observed in Co-Succinate MOF at 650 cm^−1^ and 780 cm^−1^ are attributed to CH_3_-metal groups [30], formed due to Co attachment to a methanoic group. The appearance of an additional peak for Ag/La-TMA at the spectral band of 680 cm^−1^ to 750 cm^−1^ indicates the formation of a CH_3_-Ag bond. The presence of C=O stretching in both the Co-Succinate and guest Ag@Co-Succinate confirms the existence of the carboxylic group. An extra peak is observed for Ag/La-TMA in the same spectral range of 1500 cm^−1^, signifying bond formation between Ag and the carboxylic group. Other peaks at 1300 cm^−1^ and 1580 cm^−1^ are attributed to C=O, C-O, and O-H bending vibrations of carboxylate ligands. These functional groups not involved in bond formation with guest metals may create an active site for HMI accumulation. Furthermore, after the incorporation of Apt-SH and Apt-NH onto Ag@Co-Succinate, the FTIR spectrum of Apt-SH-NH-Ag@Co-Succinate confirms the presence of thiols (SH) and amines (NH_2_). A monosubstituted layer of thiol groups is observed in weak peaks ranging from 1600 cm^−1^ to 1900 cm^−1^ [31]. Similarly, a distinct peak is observed at approximately 1360 cm^−1^, indicating the presence of amino groups. This peak indicates the successful immobilization of Apt-NH on the Ag@Co-Succinate material. The FTIR spectra clearly show the presence of amino and thiol functional groups, indicating the successful immobilization of Apt-SH and Apt-NH on the Ag@Co-Succinate material. This immobilization process plays a crucial role in customizing the material’s recognition properties for specific target analytes, thus enhancing its overall performance and selectivity in various applications, including HMI detection.

The Raman spectra for Co-Succinate and Ag@Co-Succinate are shown in Figure 1c. The 100 to 340 cm^−1^ band is attributed to metal bonding to carboxylate groups. These bands were observed for both pristine systems and Ag@Co-Succinate. Ag@Co-Succinate displayed the benzonitrile group at 950 cm^−1^ due to the utilization of nitrate-based metal salt (AgNO_3_) in the Co-Succinate modification process [32]. The one Raman active mode was observed for pristine Co-Succinate at the band of 1250 cm^−1^ to 1450 cm^−1^, which was attributed to C-H deformations. Ag@Co-Succinate also exhibited Raman mode at this band. The band discerned at 1350 cm^−1^ for Ag@Co-Succinate belongs to benzene ring stretching vibrations due to the linker–metal bonding. Except for Ag@Co-Succinate, pristine Co-Succinate showcased stretching vibrations at 870 cm^−1^ (benzene ring stretching) [33]. This exclusion of the benzene ring stretching after modification of Co-Succinate with Ag metal due to blocking all active sites showed uniform attachment of Ag to all hydrocarbons. Moreover, Ag@Co-Succinate showed fewer Raman active bands than pristine Co-Succinate, confirming the Ag metal attachment to all functional groups. Additionally, the low-intensity band at 340 cm^−1^ was attributed to the symmetric stretching of Ag-coordinated oxygen. The Raman spectrum of Apt-SH revealed distinctive peaks, with a prominent peak at approximately 1200 cm^−1^. The peak observed at 1200 cm^−1^ indicates the presence of thiol groups on the material surface, confirming the successful immobilization of Apt-SH [34]. The appearance of the 1520 cm^−1^ peak further reinforces the presence of amino groups in the immobilized Apt-NH, corresponding to the desired binding chemistry [34]. The distinct Raman spectra of Apt-SH and Apt-NH immobilized Ag@Co-Succinate materials validated the efficient incorporation of these aptamers onto the substrate. This confirmed the successful functionalization of the material surface with the desired molecular recognition elements. These findings contribute to the broader understanding of the material’s biofunctionalization and its potential applications in selective HMI sensing.

**Figure 2:**
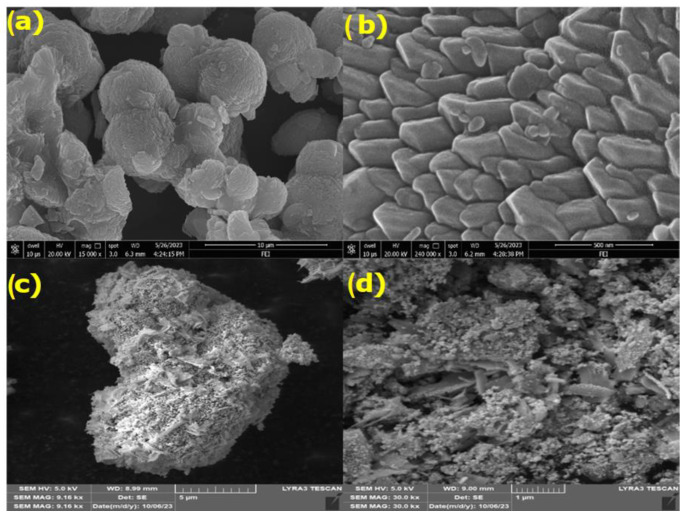
FE-SEM image of (**a**) Co-Succinate, (**b**) magnified image of Co-Succinate, (**c**) Ag@Co-Succinate, and (**d**) magnified image of Ag@Co-Succinate.

In Figure 2a, a field emission scanning electron microscopy (FE-SEM) image of Co-Succinate displays a unique and intriguing structural feature. The Co-Succinate sample has a cauliflower-like shape, with numerous interconnected spherical structures resembling florets. These structures have a complex and highly textured surface, as seen in Figure 2b. The distinctive morphology of Co-Succinate is critical because it affects the properties of the material and its potential applications. The FE-SEM image of Ag@Co-Succinate (Figure 2c,d) reveals the presence of agglomerated silver nanoparticles on the surface of Co-Succinate crystals. This observation is crucial because it indicates the successful formation of a composite and suggests the potential for unique material properties resulting from this combination. The FE-SEM images illustrate the structural attributes of Co-Succinate and Ag@Co-Succinate and provide visual evidence of the surface modifications and interplay between the materials. These observations are pivotal in shedding light on the morphological and structural changes that may influence the materials’ performance in HMI detection.

**Figure 3:**
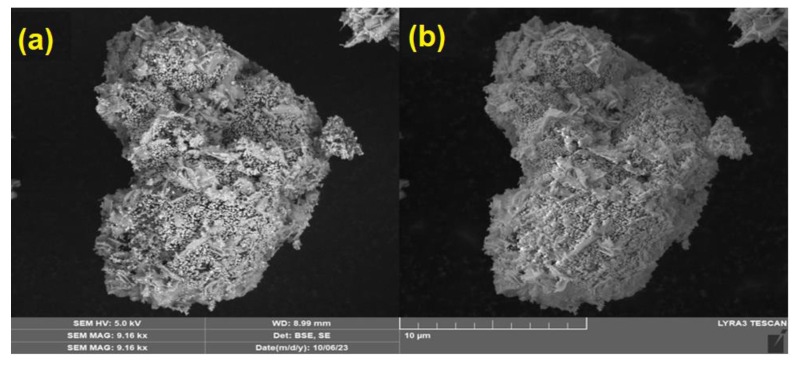
Phase contrast FE-SEM image of Ag@Co-Succinate. (**a**) Backscattered electron (BSE) scan and (**b**) normal scan.

**Figure 4:**
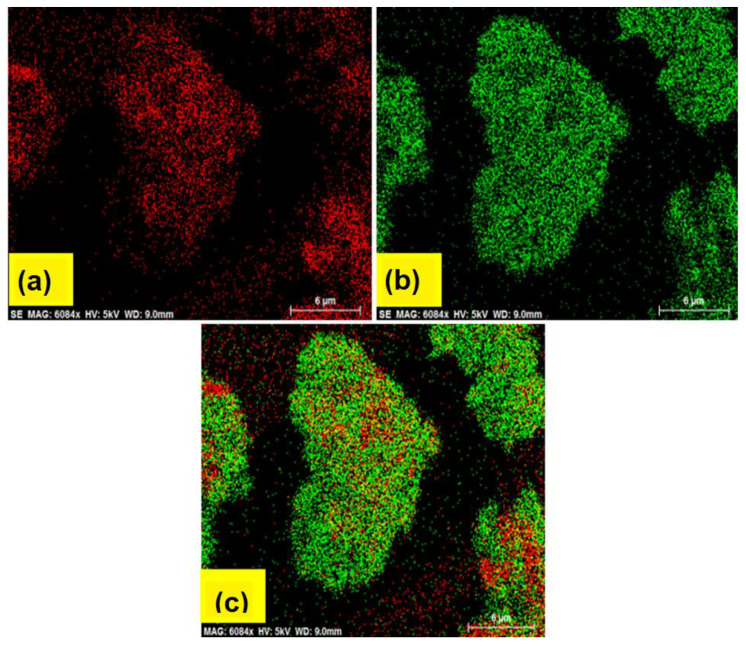
FE-SEM mapping images of Ag@Co-Succinate—(**a**) element Co, (**b**) element Ag, and (**c**) both Co and Ag elements.

The phase contrast FE-SEM image (Figure 3a,b) revealed the distribution of Ag within the Ag@Co-Succinate material. Backscattered electrons are highly sensitive to the atomic number of elements, making them an excellent tool for discerning elemental composition and density variations. The presence of Ag in the image is distinctly highlighted, allowing us to visualize the spatial arrangement of Ag nanoparticles within the Co-Succinate matrix. Figure 4 comprehensively analyzed the Ag@Co-Succinate material’s elemental distribution using FE-SEM mapping analysis. This technique allowed for the visual assessment of the spatial arrangement of Co and Ag elements in the composite material, providing insights into its structure and composition. The FE-SEM mapping image in Figure 4a clearly illustrates the distribution of Co within the Ag@Co-Succinate material. The image shows the spatial arrangement of Co atoms, highlighting the uniformity and consistent presence of cobalt throughout the material. Figure 4b showcases the Ag mapping results, offering insights into the spatial distribution of Ag within Ag@Co-Succinate. The image visually highlights the dispersion and distribution of Ag atoms, confirming the presence and arrangement of silver in the material. The combined mapping in Figure 4c reveals Co and Ag coexist within the Ag@Co-Succinate material. Importantly, it demonstrates the equal distribution of Ag throughout the material, emphasizing the successful incorporation of silver into the Co-Succinate matrix. Furthermore, the mapping results indicate the formation of strong and consistent bonds between silver and Co-Succinate. This observation is particularly significant, as it suggests the potential for enhanced material properties resulting from the well-established interaction between Ag and Co-Succinate.

#### 3.1.2. Electrochemical Characterizations of the Aptasensors

**Figure 5:**
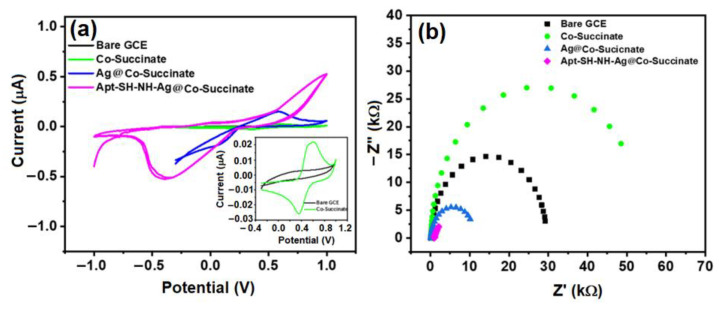
(**a**) Cyclic voltammogram. (**b**) Electrochemical impedance spectra of bare GCE, Co-Succinate, Ag@Co-Succinate, and Apt-SH-NH-Ag@Co-Succinate. The potential is vs. Ag/AgCl reference electrode.

Figure 5a shows a series of cyclic voltammograms obtained from different modified electrodes: Bare GCE, Co-Succinate-modified GCE, Ag@Co-Succinate-modified GCE, and Apt-SH-NH-Ag@Co-Succinate-modified GCE. Each modification step of the GCE resulted in unique redox peaks, with their intensities indicating the extent of electrochemical activity and redox reactions. A gradual improvement in the redox peak was observed as we progressed from bare GCE to Co-Succinate and then to Ag@Co-Succinate modifications. This enhancement indicates improved electrochemical performance and the ability of Ag@Co-Succinate to facilitate redox reactions. However, the most significant observation was seen in the cyclic voltammogram of the Apt-SH-NH-Ag@Co-Succinate-modified GCE. The redox peak was significantly enhanced, surpassing the enhancements seen in the previous modifications. This remarkable increase in the redox peak signified exceptional electrochemical activity, demonstrating the high electrochemical capabilities of the Apt-SH and Apt-NH for redox reactions of Ag@Co-Succinate. This finding is of paramount importance for the research since it suggests that the LbL immobilization of Apt-SH and Apt-NH on Ag@Co-Succinate electrodes is exceptionally well-suited for electrochemical sensing of HMIs.

Figure 5b shows the EIS analysis conducted on various modified electrodes, including bare GCE, Co-Succinate-modified GCE, Ag@Co-Succinate-modified GCE, and Apt-SH-NH-Ag@Co-Succinate-modified GCE. The EIS analysis reveals a significant trend in charge transfer resistance (R_CT_) evolution as the GCE undergoes successive modifications. Notably, the R_CT_ was consistently decreased after each modification, except in the case of the Co-Succinate modification. The initial EIS analysis of bare GCE served as a baseline, with a specific R_CT_ value, i.e., 32 kΩ. Surprisingly, the modification of GCE with Co-Succinate showed significant enhancement in R_CT_ (59 kΩ), indicating the high charge transfer characteristics of Co-Succinate due to the blocking of the active surface area of GCE. The introduction of Ag into Co-Succinate results in a decrement in the R_CT_, suggesting altered charge transfer dynamics due to presence of Ag. The most significant enhancement in R_CT_ was observed after the immobilization of Apt-SH-NH-Ag@Co-Succinate. This outcome indicated the introduction of the Apt-SH and Apt-NH, in combination with Ag@Co-Succinate, significantly affects the charge transfer resistance. The presence of both Apt-SH and Apt-NH appears to play a pivotal role in altering the charge transfer properties, potentially enhancing the sensitivity and selectivity of the modified electrode for HMI detection.

### 3.2. Electrochemical Sensing Responses of Aptasensors

**Figure 6:**
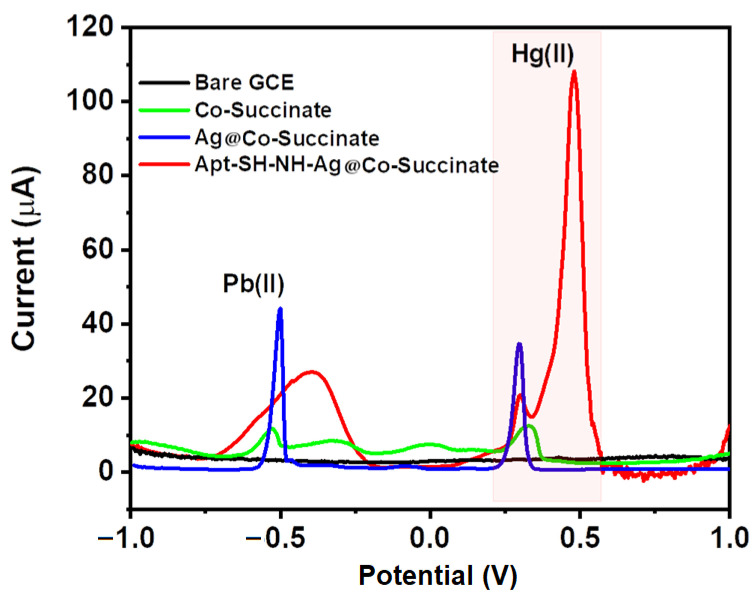
DPV responses for bare GCE, Co-Succinate, Ag@Co-Succinate, and Apt-SH-NH-Ag@Co-Succinate in PBS, pH 7.4, containing 10 nM of Hg(II), Pb(II), Cu(II), Fe(II), Zn(II), Ni(II), Cd(II), and Cr(II). The potential is vs. Ag/AgCl reference electrode.

Figure 6 shows the DPV analysis of different modified electrodes: Bare GCE, Co-Succinate-modified GCE, Ag@Co-Succinate-modified GCE, and Apt-SH-NH-Ag@Co-Succinate-modified GCE. The analysis provides crucial insights into the electrodes’ capabilities for detecting HMIs, such as Hg(II), Pb(II), Cu(II), Fe(II), Zn(II), Ni(II), Cd(II), and Cr(II). The bare GCE electrode did not show any significant analytical signal for detecting HMIs, which indicates that it has limited applicability for this purpose. Co-succinate modification yielded a weak analytical response for Pb(II) and Hg(II). Although some response was observed, it was notably feeble. The Ag@Co-Succinate modification showed a response for both Pb(II) and Hg(II), but selectivity and sensitivity issues became evident. This suggests challenges in accurately discriminating between the two HMIs and achieving optimal sensitivity. The most remarkable outcome was observed in the Apt-SH-NH-Ag@Co-Succinate modification, which displayed a highly sensitive current response for Hg(II). This distinctive behavior underscores the selectivity of Apt-SH-NH-Ag@Co-Succinate for Hg(II). Thiol aptamer (Apt-SH) and amino aptamer (Apt-NH) are crucial in enhancing sensitivity and selectivity. Moreover, these results suggest that the interference of other HMIs in the electrochemical detection of Hg(II) does not adversely affect the selectivity and sensitivity of the sensor. This finding implies that the sensor is feasible for industries where the specific target is Hg, as it maintains its ability to selectively and sensitively detect Hg(II) in the presence of other HMIs.

### 3.3. Cross-Reactivity Obtained by LbL Immobilization of Aptamers

The LbL immobilization approach is identified as a potential cause of cross-reactivity, where the Apt-SH-NH-Ag@Co-Succinate modification shows a response for Hg(II) instead of Pb(II). This observation suggests the complex interplay of aptamers and materials in the detection process, leading to selective responses. The LbL immobilization technique has indeed proven effective in many cases, enhancing the sensor’s selectivity for the intended HMIs. However, this approach can also be a double-edged sword, as this study highlights. The introduction of multiple layers, each with its specific recognition element, can lead to unexpected cross-reactivity. This research presents a case where layer-by-layer immobilization was designed for Pb(II) detection. However, to our surprise, and in contrast with the paper by Shamsipur et al. [23], the sensor exhibited a highly sensitive response to Hg(II) instead. This unexpected outcome prompted us to delve deeper into the factors contributing to this cross-reactivity. This investigation uncovers several factors that contribute to the observed cross-reactivity. The interaction between different aptamers in the LbL immobilization process can lead to unexpected recognition patterns. The properties of the substrate and materials used in the immobilization process can influence the binding affinities of the recognition elements. The electrochemical mechanisms involved in HMI detection can be complex, with multiple variables affecting the sensor’s response.

Herein, both the aptamers (Apt-SH and Apt-NH) were developed to detect Pb(II) instead of Hg(II). The ligand exchange reaction occurs between the thiol functional group and amino functional groups. Due to this, the bonds between the metallic entities present in the Ag@Co-Succinate surface and an electron-donating end group ligand molecule, such as thiol and amine, undergo dynamic binding and unbinding processes [35,36]. Moreover, the ligand molecules on the surface can be exchanged by other ligands, possibly providing new properties or functionality to the particles. This may lead to aggregation of the particles. Such aggregation leads to disturbing the specific binding capacity of the aptamers to bind targeted HMIs.

### 3.4. DPV Response for Hg(II), Calibration Curve and Repeatability

**Figure 7:**
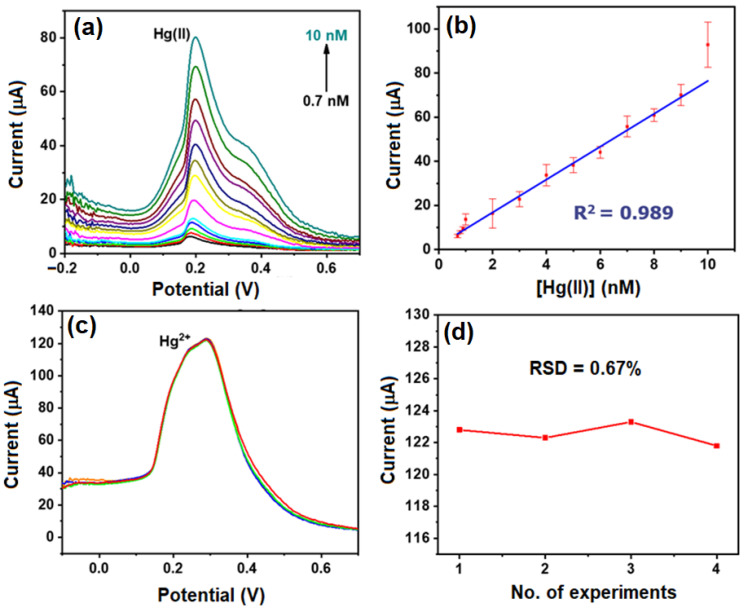
(**a**) DPV response of Apt-SH-NH-Ag@Co-Succinate in PBS containing Hg(II) concentration ranging from 0.7 nM to 10 nM, (**b**) calibration plot including error bars for each concentration range, (**c**) repeatability experiments for Apt-SH-NH-Ag@Co-Succinate in PBS containing 10 nM Hg(II), and (**d**) current response against number of experiments. The potential is vs. Ag/AgCl reference electrode.

Figure 7a shows the DPV response of Apt-SH-NH-Ag@Co-Succinate towards Hg(II) ranging from 0.7 nM to 10 nM. The presence of Hg(II) improves the charge transfer capabilities of Ag@Co-Succinate, as evidenced by a steady and dramatic increase in the current responses, as shown in Figure 7a when Hg(II) concentration is raised. Calibration plots (Figure 7b) show the linearity of the obtained analytical current responses. The minimum deviation in the error bar for each of the concentration levels of Hg(II) offers the accuracy and stability of the developed Apt-SH-NH-Ag@Co-Succinate sensor. Sensitivity was found to be 7.45 µA/nM with R^2^ = 0.989. The limit of detection (LOD) has been calculated according to the formula 3σ/s, where σ is the standard deviation of current responses obtained at lower HMIs concentration, and s is the slope of the calibration curve. The determined LOD = 0.3 nM was far below the maximum concentration level (MCL) suggested by the US-EPA [2]. Four independent experimental trials yielded consistent findings at 10 nM Hg(II) concentrations. Figure 7c shows the repeatable differential current overlapping signals. This confirmed the current output after a repeatable sort of experiment at a low molar concentration of Hg(II), as shown in Figure 7d.

The comparison between the electrochemical sensor based on aptamer-modified Ag@Co-Succinate (present work) and earlier reported aptamer-modified sensors with other materials for the detection of HMIs is illustrated in Table 1. It can be seen that the aptamer-modified Ag@Co-Succinate (present work) is better in terms of the range of detection and LOD.

### 3.5. Binding mechanism of DNA Aptamers and Hg (II)

**Scheme 2:**
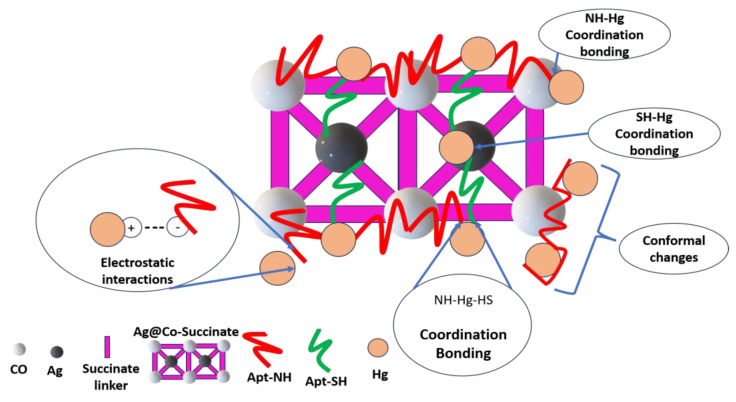
Possible binding mechanism of DNA aptamers with Hg(II).

The binding mechanism of DNA aptamers (Apt-SH and Apt-NH) with Hg(II) involves a series of coordinated interactions that contribute to the formation of a stable aptamer–metal complex. As depicted in Scheme 2, the interactions between aptamers and Hg(II) can be broadly categorized into coordination bonds, electrostatic interactions, and conformational changes in the aptamers upon binding. The thiol group in Apt-SH plays a crucial role in binding with Hg(II) ions. Mercury ions have a high affinity for thiol groups, forming strong coordination bonds. The interaction typically involves the displacement of water molecules from the Hg(II) coordination sphere by the sulfur atom of the thiol group.

In Apt-NH, the amino groups contribute to the binding mechanism through coordination with Hg(II). The lone pair of electrons on the amino groups form coordination bonds with the metal ion, stabilizing the aptamer–metal complex. Both Apt-SH and Apt-NH may engage in electrostatic interactions with Hg(II) ions. The negatively charged phosphate backbone of DNA and the positively charged metal ions create favorable electrostatic attractions. These interactions can enhance the overall stability of the aptamer–metal complex. The synergistic effect of these interactions leads to the formation of a stable aptamer–metal complex, enabling selective and sensitive detection of Hg(II) ions.

## 4. Conclusions

This research aimed to improve HMI detection methods by exploring innovative strategies such as LbL immobilization. The use of LbL immobilization showed the potential to enhance the sensitivity and selectivity of HMI detection. The process involved sequential attachment of aptamers (Apt-SH and Apt-NH) and Ag@Co-Succinate on the electrode surface, which allowed for tailored responses and introduced a dynamic dimension to the electrochemical sensing landscape. During the investigation, unexpected cross-reactivity was observed—instead of the anticipated response to Pb(II), the system exhibited sensitivity to Hg(II). This unexpected outcome challenges traditional assumptions and emphasizes the need for a deeper understanding of the factors influencing aptamer–material interactions. Before incorporating the effects of LbL immobilization, Ag@Co-Succinate MOF was used as a new electrode modifier for detecting HMIs as an electrochemical sensor. This study highlighted the crucial role of aptamers in determining selectivity. The inclusion of thiol aptamer (Apt-SH) and amino aptamer (Apt-NH) significantly influenced the system’s response, with Apt-SH-NH-Ag@Co-Succinate displaying heightened sensitivity (7.45 µA/nM) and selectivity towards Hg(II) with an unprecedented detection limit of 0.3 nM. Moreover, the proposed sensor exhibited a repeatable analytical response with a minimum RSD of 0.67%. This emphasizes the complex interplay between aptamer sequences and the target ions.

The practical applications of Hg(II) detection presented in this work extend to diverse real-world environments, including environmental monitoring in industrial settings, water quality assessment, and compliance monitoring in regulatory contexts. Our sensor’s selectivity and sensitivity make it a valuable tool for rapid, on-site detection of mercury contamination, aiding in early intervention and effective environmental management. Additionally, its portability and ease of use position it as a potential asset for fieldwork and emergency response scenarios, contributing to the broader goal of safeguarding human health and environmental well-being.

## Figures and Tables

**Table 1:** Comparison between the electrochemical sensor based on aptamer-modified Ag@Co-Succinate (present work) and earlier reported aptamer-modified sensors with other materials for detection of HMIs.

Materials for Aptamer Immobilization	HMI Detected	Range of Detection	LOD	Ref.
Ag-Au alloy NPs	AS(III)	0.01–10 µg/L	3 ng/L	[37]
Ag-Au alloy NPs	Pb(II)	0.01–10 µg/L	0.8 µM	[38]
T33/TOTO-3	Pb(II) Hg(II)	3–50 nM 25–500 nM	1 nM 10 nM	[39]
Au nanoparticles	Hg(II)	1 nM–100 µM	0.6 nM	[40]
Au nanoparticles	Hg(II)	0.02–1 µM	16 nM	[41]
Graphene/Fe_3_O_4_-AuNP	Pb(II)	1–300 ng/mL	0.63 ng/mL	[42]
SiO_2_@AuNPs	Hg(II)	10 nM–1 mM	10 nM	[43]
ZIF-8 MOF	Pb(II)	0.1–10 µg/L	0.096 µg/L	[44]
Ag@Co-Succinate	Hg(II)	0.7–10 nM	0.3 nM	This work

## Data Availability

Data are contained within the article.
